# Supplementary material for: Prediction of the Formation of Reactive Metabolites by A Novel Classifier Approach Based on Enrichment Factor Optimization (EFO) as Implemented in the VEGA Program
Source: Molecules. 2018 Nov 13;23(11):2955. doi: 10.3390/molecules23112955 (PMC6278469; doi:10.3390/molecules23112955)
Supplement: Supplementary file 1 [file molecules-23-02955-s001.zip › Scheme_S1.docx]

**Supporting Information**

**Prediction of the formation of reactive metabolites by a novel classifier approach based on enrichment factor optimization (EFO) as implemented in the VEGA program**

Angelica Mazzolari, Giulio Vistoli, Bernard Testa, Alessandro Pedretti

**Scheme S1:** Pseudocode for the here proposed EFO algorithm

**program EFO**

input: Matrix P(m,d) of m rows (molecules) and d columns (descriptors)

Vector T(m) of boolean values indicating the class of each molecule

Constant N as descriptors to be used in the output model (e.g. 2)

Constant CS as cluster size (e.g. 100)

output: Vector L() of structures of calculated models:

L {

Subset D of indexes of the descriptors

Vector W(N) of coefficients of linear function

Real QM quality of the model

}

**** Selection of the most significant descriptors ****

for each descriptor d

if CoefficientOfVariation(P(.,d)) < 0.01

Exclude the descriptor d

endif

endfor

**** Generation of the models ****

new Vector W(N) **** Coefficients of the function to minimize ****

a = 0

for each subset D of descriptors of cardinality N

a = a + 1

La.D = D

for i = 1 to N

W_i_ = Random()

endfor

**** Minimize the separation function ****

(La.W, T0) = HookeJeevesMinimizer(La.W, N, f, P, m, d, T)

**** Cluster analysis ****

CN = RoundUp(m / CS) **** Number of clusters ****

new Vector CL(CN) **** Clusters ****

for i = 1 to CN

CL_i_ = 0

endfor

**** Count the molecules of true class of each cluster ****

i = 1 **** Cluster index ****

k = 1 **** Cluster element ****

for j = 1 to m

if (k > CS)

k = 0

i = i + 1

endif

if (T_j_ = true)

CL_i_ = CL_i_ + 1

k = k + 1

endif

endfor

**** Count the molecules of true class in the top 1% ****

TC1 = 0 **** Number of molecules of true class in the top 1% ****

for j = 1 to (m * 0.01)

if T_j_ = true

TC1 = TC1 + 1

endif

endfor

**** Calculate the quality of the model (QM) ****

La.QM = TC1 * Kurtosis(CS, CN) * Skewness(CS, CN)

if La.QM > 0 and (Kurtosis(CS, CN) < 0 or Skewness(CS, CN) < 0)

La.QM = -La.QM

endif

endfor

**** Sort the calculated models ****

sort L in ascending order according to QM

**endprogram**

**** Function to minimize ****

**Function f(Vector W, N, Matrix P, m, d, Vector T)**

input: Vector W(N) of coefficients to minimize

Matrix P(m,d) of m rows (molecules) and d columns (descriptors)

Vector T(m) of boolean values of the class of each molecule

output: Vector W(N) of minimized coefficients

Real C as model score

Vector T0(m) of classes sorted by S

new Vector S(m) **** Scores of each molecule ****

for each molecule j

Sj = 0

for i = 1 to N

S_j_ = S_j_ + W_i_ * P_j,i_

endfor

endfor

new Vector T0(m) **** Sorted vector of classes ****

T0 = sort T in ascending order according to S

C = 0 **** Score of the model ****

for each molecule j

if T0_j_ = true

C = C + S_j_

endif

endfor

return(W, C, T0)

**endfunction**

**** Other functions ****

function CoefficientOfVariation(Vector V, N)

Calculate the coefficient of variation of the vector V of N elements.

function HookeJeevesMinimizer(Vector W, N, function f, Matrix P, m, d, Vector T)

Minimize the N coeffocients W of the function f, P, m, d and T are parameters

to pass to the function f.

function Kurtosis(Vector C, N)

Calculate the kurtosis of a cluster distribution C of N elements.

function Random()

Generate a random number.

function RoundUp(Real N)

Round up N to integer.

function Skewness(Vector C, N)

Calculate the Skewness of a cluster distribution C of N elements.
